# Physical Exploration and Diagnosis of Diseases Affecting the Respiratory Organs

**Published:** 1856-05

**Authors:** 


ART. IV.—*Physical Exploration and Diagnosis of Diseases affecting the Respiratory Organs.* By AUSTIN FLINT, M. D., Professor of the Theory and Practice of Medicine in the University of Louisville; Honorary Member of the Medical Society of Virginia, and of the Kentucky State Medical Society. Philadelphia: Blanchard & Lea. 1856.

Turning from the title-page of this volume, the reader will pause a moment to notice the dedication to Professors Coventry, Lee, White, Hamilton, Hadley, Palmer, Dalton, and Moore; "with whom the author was formerly associated in the University of Buffalo, and to his medical friends in the city of Buffalo"—a pleasant reminder to his old *confreres*, that, though no longer a colleague, he still remembers the school with whose origin and progress he was so intimately connected, and in which he laid the foundation for a solid and permanent reputation; and a token of good will to those others with whom he has been at once a competitor and a friend.

In undertaking a work on Physical Diagnosis, in addition to his previous valuable monographs on Continued Fever and other subjects of practical medicine, Dr. Flint has not performed a task of supererogation. Almost the only comprehensive work on the subject in our language was that of Walshe, and even it, like those of Skoda, Swett, Bowditch and others, admirable as they are in many particulars, left a vacuum for another to fill. Some attempted too much, while others confined their efforts to too narrow a compass.

The design of the work under notice, is to give a full and comprehensive treatise on all matters pertaining to the diagnosis of diseases of the respiratory organs. The physical exploration of the heart was omitted as a subject large enough by itself, while pathology and practice are also omitted for a similar reason. Confined, then, to this one subject, it is, on that, full without diffusenees or tediousness of detail, and may be fairly considered as a representation of the present state of knowledge in this branch of medical science. Its six hundred ample pages may be said to exhaust the subject for the present.

There is a distinctive feature in the book which we are glad to notice. Up to the present time, no observer had taken the pains carefully to compare and analyze the sounds of the healthy chest. They have been assumed from a principle of general analogy, or have taken as their standard some portion of the lung supposed, in the individual case, to be healthy. It is obvious, that, while this imperfect method may tolerably well subserve the purposes of diagnosis in acute disease, yet in many chronic diseases, liable to be confounded with an incipient phthisis, it is vitally necessary to the accuracy of the diagnosis, as well as to the safety of the patient, that the law of health should be known. A case of suspected phthisis presents itself, and the observer finds the expiratory murmur prolonged, with slight comparative dullness on percussion on the right side. These are early incipient signs of tubercle, but they lose much of their value when it is known that this is, in the majority of instances, the law of heal'h. On the other hand, let these same signs be found upon the left side, in contradiction of that law, and they become possessed of corresponding importance.

We might further illustrate the importance of this consideration, but prefer to state that the laws here deduced as to the sounds of health, are derived from a careful examination of twenty-four healthy chests, male and female. The number appears, at first glance, insufficient; but when it is recollected that an *accurate* exploration of this number, with a proper written entry of results, is really a prolonged, tedious and difficult task; that no other author has undertaken anything of the kind; that the subjects were carefully selected, and that they can hardly have failed to present all varieties of healthy sounds; the objection disappears. We introduce here a few of the results of these explorations:

"It thus appears that in the majority of persons in health, having wellformed chests, there is not an absolute equality in the resonance existing at the summit of the chest in front on the two sides. It appears, also, that, as a general rule, the disparity consists in a greater degree of resonance, more vesicular quality, and, relatively, lowness of pitch, on the left side. The tympanitic quality is occasionally found on one side, which may be either the left or the right. The practical bearing of these facts will appear hereafter; the facts rest on observation, and are independent of any explanation that may be offered. Theoretically, in view of the greater capacity of the right chest, it would seem, perhaps, more reasonable that the difference between the two sides should be the reverse of that which is found to exist. The larger development of the right pectoral muscle, in consequence of the greater use of the right upper extremity, may account for the fact in some instances, but the disparity exists in cases in which there is no apparent difference in the muscular covering, in this situation. Possibly the different physical conditions at the base of the thorax may afford an explanation. On the right side the lungs repose, with the diaphragm intervening, on the liver, which occupies the whole of the base on that side. The presence of this solid viscus may slightly deaden the sound. On the left side below the lung is situated the stomach, frequently more or less distended with gas, and the effects of this, it may be supposed, is to increase the sonorousness on that side, even at the summit, independent of the transmission of the tympanitic gastric sound which is sometimes observed."

Independent of any theory connected with their explanation, the bare facts mentioned here are important, as leading us to a more favorable diagnosis in case of slightly impaired resonance or vesicular quality on the right side, and an equally unfavorable one should the left side be the one presenting the signs. Other facts connected with auscultatory sounds, tending in the same direction, are presented in another part of the volume, but as they have an immediate bearing upon the diagnosis of incipient consumption, we insert them here:

*Comparison of Right and Left Infra-clavicular Regions. Whole number of examinations thirty-four.*

INSPIRATORY SOUND.

| *Right.* | *Left.* |
|---|---|
| Greater intensity in 1 case. | Greater intensity in 15 cases. |
| Vesicular quality more marked in no case. | Vesicular quality more marked in 14 cases. |
| Higher pitch of sound in 12 of 19 examinations. | Higher pitch of sound in no case. |

EXPIRATORY SOUND.

| *Right.* | *Left.* |
|---|---|
| Present on this side, and not on left side, in 3 cases. | Present on this side, and not on the right side, in no case. |
| More intense on this side in 2 cases. | More intense on this side in no case. |
| Prolonged in several cases. | Prolonged in none. |
| An interval between the sounds of inspiration and expiration in several cases. | The two sounds continuous. |
| Pitch higher than that of the inspiratory sound in 11 instances. | Pitch higher in 1 instance. |
| Pitch lower than that of inspiration in 4 instances. | Pitch lower in 10 instances. |

We have thus a most important series of facts, deduced from these examinations of healthy chests, bearing upon the presence of disease in the regions always regarded as the most liable to the invasion of tubercle, viz., the infra-clavicular region.

Within certain limits, all signs of disease are reached by comparison of the two sides, one being assumed as the type of health. Now here upon the right side, in the infra-clavicular region, we have comparative dullness on percussion, less vesicular quality, less intensity of inspiratory sound, with prolongation and higher pitch of the expiratory sound, in the majority of cases. All these are, *when properly correlated*, signs of incipient tuberculosis; while viewed as probable normal variations, they may save us many a serious error in diagnosis. In this connection we cannot better enforce the necessity of attention to these normal differences, than by a brief quotation from Chapter IX, on the "Correlation of Physical Signs." It is here, perhaps, more than in any other point, that practitioners fail in diagnosis. One sign absorbs the attention, becomes disconnected with its associates, and acquires an undue importance.

"Very few, if any, of the physical indications of pulmonary disease are pathognomonic. As a general rule, their diagnostic importance is in a great measure derived from union with each other; and this aggregation of different signs, while it is often essential to diagnosis, always renders it much more exact and positive. A group of signs, no one of which by itself would be reliable, sometimes points to the nature and seat of a disease with greater precision than the most distinctive characteristic taken singly. To cite an illustration of this truth, the existence of tuberculous disease may be established by a series of phenomena, each of which, without the others, would possess trivial importance; but, collectively, they render the diagnosis as complete as possible. On the other hand, let one of the most significant of the physical signs be selected, for example metalic tinkling: guided by it exclusively, there would be a liability to error, for, although in the immense majority of the cases in which it is marked, it indicates pneumo-hydrothorax' it may occur in connection with a large pulmonary excavation, and is simulated by sounds produced within the stomach. The accumulated evidence, in the first instance, overbalances the weight to be attached to the single sign, for reasons not unlike those which give to an abundance of circumstantial proof in courts of law greater force than belongs to the strongest direct testimony of a single individual. Again, not only are physical signs individually insufficient as diagnostic criteria, but the same sign may be incident to different affections. The bronchial respiration, for instance, belongs equally to the semeiological history of pneumonitis and tuberculosis. Absence of respiratory sound occurs in cases of emphysema and in cases of pleurisy,—two very dissimilar forms of disease. The significance of particular signs, in such instances, depends in a great measure on the combinations in which they are found. Thus, absence of the respiratory murmur in emphysema is associated with an abnormal clearness of percussion-resonance; on the other hand, in pleurisy, it is accompanied by flatness on percussion. The significance of the respiratory sign in these two instances is borrowed from the coëxisting signs, the latter, it will be observed, being exactly opposite in their character."

Part I of the work closes with this chapter on the Correlation of Physical Signs, some 350 pages having been devoted to a discussion of anatomical points, topographical division of the chest, to the definitions necessary to the subject, to Percussion in Health and Disease, to Auscultation in each of these conditions, to Succussion, Inspection, Mensuration, Palpitation, and to a recapitulatory statement of different physical signs.

With Part II we commence the "Diagnosis of Diseases affecting the Respiratory Organs."

Passing over the acute form of bronchitis, we extract the author's remarks on the differential diagnosis of capillary bronchitis and lobular pneumonia:

"In the lobular form of pneumonitis, as has been already stated, and as will appear more fully hereafter, the characteristic physical signs, as well as certain symptoms pertaining to lobar inflammation of the pulmonary parenchyma, are frequently wanting. The crepitant râle, the bronchial respiration and bronchophony, are often not discoverable. The matter of expectoration in young children is swallowed. In view of these facts, how is the differential diagnosis to be made? The following are the chief points of distinction. General capillary bronchitis, as a rule, is a graver affection than lobular pneumonitis; the respirations are more frequent; the asphyxiating effects are greater, and the symptoms representing these effects, viz, dyspnœa, restlessness, lividity, in a corresponding degree more marked. In fatal cases, the career of the disease is more rapid. With reference to physical signs, one source of difficulty is the incompleteness of the explorations with which the physician must be content in examining young children. With care and perseverance the characteristic phenomena of pneumonitis may, in some cases, be discovered. In addition to the auscultatory phenomena just mentioned, if the number of lobules consolidated by inflammation be considerably more numerous on one side than on the other, relative dullness on percussion may be apparent. But the same result will follow collapse of a great number of lobules on one side from bronchial obstruction. The sub-crepitant râle belongs to both affections, but in lobular pneumonitis it is limited in its seat to the minute tubes in immediate relation to the inflamed lobules, while in general capillary bronchitis the physical conditions for the production of the sound exist everywhere throughout the lungs. In the latter affection, therefore, the sub-crepitant râle is diffused over the whole surface of the chest; and in the former it is limited to certain portions. This is the most distinctive evidence to be obtained by physical exploration, provided the positive signs of pneumonitis are not to be discovered. In the instances in which the signs are appreciable, the diagnosis is, of course, established."

The author adds that, with all due attention to these distinctive points, a positive diagnosis may still be impossible; and remarks that this well accords with the existing confusion in our ideas of the pathology of the two affections. It seems that here is a point yet to be settled, viz, whether lobular pneumonia is anything more than collapse of lung from capillary bronchitis. To our mind there is yet another problem! Is capillary bronchitis necessary to collapse of lung? The discussion of these questions is unfortunately not included in the plan of this volume.

The signs of pseudo-membranous, chronic and secondary bronchitis, are next considered with great clearness and precision. Following them we have a chapter on "Dilatation and Contraction of the Bronchial Tubes—Pertussis—Asthma."

Under the head of pneumonia, three divisions are made, viz., Lobar Pneumonia as found in the adult; Lobular Pneumonia as it exists in children; and, finally, Chronic Pneumonia.

At the outset, the author states a point which has recently been disputed in this locality; that in lobar pneumonia the inflammation is, in a vast majority of cases, diffused through the entire lobe. Of the varieties of primary lobar pneumonia, he acknowledges the simple acute pneumonitis; speaks of typhoid pneumonia as sometimes a development of the simple form, and sometimes (perhaps very often) an intercurrent inflammation in the course of continued fever, though the fever may fail to be recognized. Of the term "bilious pneumonia" he says that "in its application to cases complicated with icterus, it has an obvious significance," but doubts its propriety in those cases in which the evidence of disorder of the liver is less marked.

*Physical Signs.* Prof. Flint agrees with most practical authors, but differs from Skoda, in stating that percussion resonance is impaired in the stage of engorgement. He gives a case in which death occurred from other causes than pneumonia, and a post-mortem was made. The lung was found in the stage of engorgement, while the percussion signs, before death, had been clearly deficient in resonance. An instance entirely similar occurred to ourself during the past winter. The occurrence of tympanitic resonance above the lobe affected, so common in the second stage of pneumonia, and often heard in chronic pleurisy, is accounted for in part by considering it as a sound transmitted from the stomach or intestines. The rapid fluctuations occurring in some cases are, however, assigned to the bronchial tubes and the variable amount of secretion in them.

Under the head of Lobular Pneumonia, Dr. Flint includes broncho-pneumonia; imperfect expansion (atelectasis); and collapse of pulmonary lobules. This is done, however, only so far as diagnosis is concerned; the actual distinctions which exist are recognized, and very clearly defined. Indeed, the rapid but brief description of these important distinctions is remarkable for its lucidity.

Of Chronic Pneumonia but little is said. The existence of the disease, doubted by some, is admitted by the auther; but cases of it are stated to be exceedingly rare.

The remainder of the volume is devoted to the signs of Emphysema, Tuberculosis, Bronchial Phthisis, Pulmonary Œdema, Gangrene of the Lungs, Pulmonary Apoplexy, Cancer of the Lungs, Cancer of the Mediastinum, Acute and Chronic Pleuritis, Empyema, Hydrothorax, Pneumothorax, Pneumo-Hydrothorax, Neuralgia, Diagphragmatic Hernia, Diseases affecting the Trachea and Larynx, and an appendix on the Pitch of the Whispering Souffle over Pulmonary Excavations.

We would gladly follow our author through these various subjects, but both time and space forbid. We can only state our general impression of the high value of this work, and cordially recommend it to all. We regard it in point both of arrangment, and of the marked ability of its treatment of the subjects, as destined to take the first rank in works of this class. So far as our information extends it has at present no equal.

To the practitioner, as well as the student, it will be invaluable in clearing up the diagnosis of doubtful cases, and in shedding light upon difficult phenomena.

For sale by PHINNEY & Co.

